# Sarcoidosis mimicking lymphoma on positron emission tomography-computed tomography in two patients treated for lymphoma: two case reports

**DOI:** 10.4076/1752-1947-3-7306

**Published:** 2009-06-23

**Authors:** Ozden Ozer, Ahmet Emre Eskazan, M Cem Ar, Hüseyin Beköz, Fehmi Tabak, Gül Ongen, Burhan Ferhanoglu

**Affiliations:** 1 Istanbul Pathology Group, Istanbul, Turkey; 2 Department of Internal Medicine, Division of Haematology, Cerrahpasa Medical Faculty, Istanbul University, Kocamustafapaşa, Istanbul, Turkey; 3 Florence Nightingale Gayrettepe Hospital, Istanbul, Turkey; 4 Department of Infectious Diseases and Clinical Microbiology, Cerrahpasa Medical Faculty, Istanbul University, Istanbul, Turkey; 5 Department of Chest Medicine, Cerrahpasa Medical Faculty, Istanbul University, Istanbul, Turkey

## Abstract

**Introduction:**

Sarcoidosis is a granulomatous disease that mostly involves the lungs. Its association with malignancies has been well documented. Several mechanisms have been proposed that may underlie this concurrence including triggering tumour antigens and defective cellular immunity.

**Case presentations:**

We briefly review the literature on malignancy associated sarcoidosis and report two female lymphoma patients of 49 and 56 years of age who, during their course of disease, developed sarcoidosis that was misinterpreted as a lymphoma relapse on positron emission tomography-computed tomography.

**Conclusion:**

We hypothesise that T cell dysfunction and exposure to tumour associated antigens might be the underlying mechanisms of development of sarcoidosis in patients with lymphoma. Positron emission tomography-positive lesions do not always indicate malignancy and therefore a tissue biopsy is always mandatory to confirm the diagnosis.

## Introduction

Sarcoidosis is an uncommon granulomatous disease primarily manifesting itself with pulmonary involvement. Any organ, including the eye, central nervous system, and even gonads can be involved. Systemic sarcoidosis, in particular localised sarcoidal reaction, is well documented in association with malignancies. We report two patients who developed sarcoidal reactions in association with lymphoma.

### Case 1

**Figure 1:**
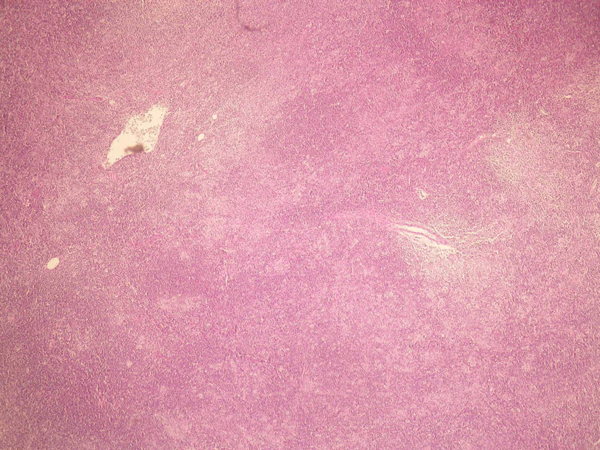
**The low power view of the lymph node shows an effaced nodal architecture**. There is a mottled appearance due to lighter staining histiocyte-rich areas and darker staining sheets of small mature appearing lymphocytes.

**Figure 2:**
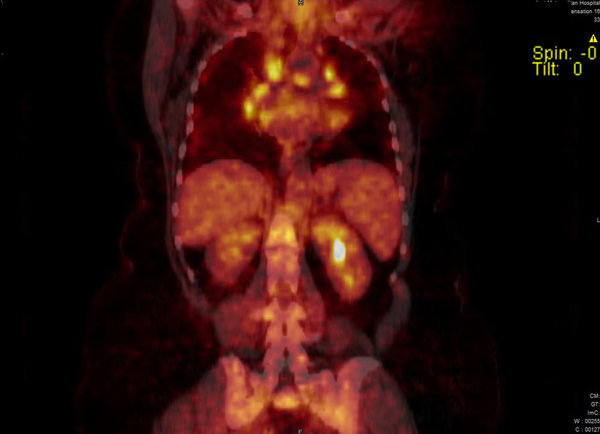
**Positron emission tomography-computed tomography image showing hypermetabolic mediastinal lymph nodes**.

**Figure 3:**
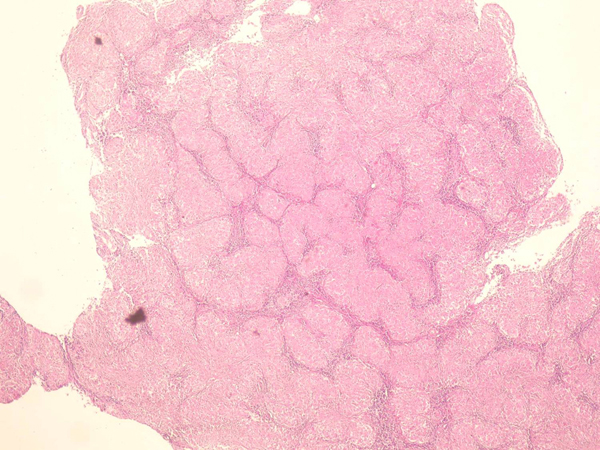
**The mediastinal lymph node, excised after completion of doxorubicin, bleomycin, vinblastine, and dacarbazine therapy, was significant for complete architectural effacement by tightly packed granulomas**.

A 56-year-old woman presented with enlarged right cervical lymph nodes (LN) that failed to regress following appropriate treatment with antibiotics. The largest LN measured 3.8 cm in diameter. A fine needle aspiration of the LN was highly suspicious for Hodgkin's lymphoma (HL). The excisional biopsy of the LN revealed complete architectural effacement with scattered Reed-Sternberg (RS) cells, histiocytes and eosinophils (Figure 1). RS cells were CD20-, CD45- and CD30+. Fascin, which is not specific for HL, but its negativity would make a diagnosis of HL doubtful, stained most of the RS cells. A diagnosis was made of "HL, classical type, mixed cellularity subtype". Positron emission tomography-computed tomography (PET-CT) revealed additional LNs in the cervical and supraclavicular region. Bone marrow biopsy was negative for lymphoma. Serologic tests for hepatitis B, C and HIV were negative. The patient was, thus, staged as stage I, non-bulky HL, unfavourable, due to her age being over 50. Combined therapy with four cycles of doxorubicin, bleomycin, vinblastine, and dacarbazine (ABVD) chemotherapy followed by involved field irradiation was scheduled. During chemotherapy, our patient experienced respiratory difficulties which was not associated with infection. Two control PET-CTs taken at the end of four cycles of ABVD and at the end of 30.6 Gy/17 fractionated radiotherapy to the right neck and supraclavicular region showed complete remission. However, a routine third PET-CT performed 3 months following completion of chemoradiotherapy, revealed several fluorodeoxyglucose (FDG) enhancing mediastinal LNs (Figure 2). There was also FDG-enhancing thickening of the pleura in the left lung laterobasal segment and an ill-defined increase in parenchymal density. The findings were interpreted as strongly likely to be recurrent lymphoma and the possibility of high-dose chemotherapy with autologous stem cell transplantation was discussed with the patient. To confirm the diagnosis, a mediastinoscopic LN biopsy was performed. The LN architecture was completely effaced by tightly packed non-caseating granulomas, indicating sarcoidosis (Figure 3). There was no histological evidence of HL. The patient was then referred to a chest physician for consultation. A tuberculin test was negative. Carbon dioxide diffusion was slightly reduced. Based on the clinical and radiological findings, the patient was diagnosed with sarcoidosis and steroid therapy was initiated.

### Case 2

The second patient was a 49-year-old woman who was diagnosed with diffuse large B cell lymphoma of germinal centre origin in an axillary LN biopsy (CD20+, bcl-6+, CD10+). Additional small LNs of 1.2 cm and 1.5 cm diameter were identified in the thoracic and abdominal cavities. There was neither bone marrow involvement nor hepatosplenomegaly. Serologic tests for hepatitis B, C and HIV were negative. She was staged as IIIA and put on rituximab, cyclophosphamide, doxorubicin, vincristine, prednisolone (R-CHOP) chemotherapy. After four courses of R-CHOP, there was regression in the enlarged peripheral LNs but not in those of the abdomen and mediastinum. The therapy was extended to a total of eight courses with no response. A PET-CT confirmed the hypermetabolic status of the non-regressing LNs as likely indicating persistent lymphoma. A mediastinoscopic LN biopsy was performed to confirm the diagnosis. However, it revealed diffuse replacement by sarcoidal type non-necrotizing granulomas with no histological evidence of lymphoma. The patient was consequently referred to a chest physician for further evaluation. No therapy for sarcoidosis was instituted since the patient was asymptomatic and had no pulmonary parenchymal involvement.

## Discussion

These two cases clearly illustrate that not every 'PET-positive' lesion represents malignancy and a tissue biopsy is mandatory to confirm the diagnosis. Sarcoidosis is a poorly understood idiopathic disease which is classically defined as the occurrence of non-caseating granulomatous inflammation in the absence other conditions such as infection and malignancy. There are, however, some cases of sarcoidosis reported to occur in association with a broad spectrum of diseases, ranging from Hodgkin's and non-Hodgkin's lymphomas, germ cell tumours, carcinomas to autoimmune diseases and therapy with immuno-modulatory drugs. Therefore, some authors prefer to use the terms 'sarcoid-like lymphadenopathy' or 'sarcoidal reaction' instead of 'sarcoidosis' to describe lymph node enlargement with non-caseating granulomas in the context of malignancy or infection [1].

Sarcoidosis is considered as a type of florid cell mediated immune reaction by histiocytes. In vivo/vitro anergy, corresponding to cytotoxic T cell defect and increased T helper cell activity, has been reported to be consistent with defective cellular immunity [2]. Increased T helper cell activity may represent a positive feedback loop, which under normal circumstances would be silenced by signals from activated cytotoxic T cells [3]. In this instance, the exuberant histiocytic reaction may well represent a physiologic substitution to the T cell defect, mediated by increased T helper cell activity. The extent of this reaction may be determined by additional factors, most importantly a triggering stimulus, which may be exogenous, tumour or self antigens.

Classical signs of sarcoidosis start in the pulmonary system. It is interesting that well recognised examples of silicosis may be indistinguishable from sarcoidosis on light microscopy only, further implicating the role of antigenic stimuli that may trigger sarcoidosis. Therefore, while some cases diagnosed as sarcoidosis may actually be pneumoconiosis, a typical sarcoidosis may represent interplay between the abnormal host immune system and triggering antigens of specific immunogenic potential [4]. This overlap suggests that the T cell defect shown in sarcoidosis is contributory but not essential in the formation of florid granulomas.

When we search through the sarcoidosis-malignancy association in the literature, sarcoidosis plays the most frequent association with HL among haematopoietic malignancies and with germ cell tumours among non-haematopoietic malignancies [5,6]. This predilection endows a critical role to specific tumour antigens in the pathogenesis of sarcoidosis, suggesting that HL and seminoma antigens are more 'sarcoidogenic' than others. Localised sarcoidal reactions in lymph nodes draining the site of the malignancy are the most frequent if not exclusive presentation in malignancies. The temporal relation of malignancies and sarcoidosis varies; they may precede, follow or co-exist with malignancies [5]. Systemic sarcoidosis increases the risk of HL. This is likely due to the underlying immune deficiency permitting uncontrolled Epstein-Barr virus (EBV) expansion, an oncogenic virus frequently indicated in the aetiology of HL. There are rare examples of sarcoidosis occurring after successfully treated HL, similar to one of our patients [7].

Systemic sarcoidosis or localised reactions are also seen after therapy with immuno-modulator agents. Interferon therapy for Hepatitis C infection may cause sarcoidosis with pulmonary and/or cutaneous involvement [8]. While cessation of therapy resulted in regression in drug induced cases, in patients with pre-existing sarcoidosis, interferon had caused exacerbation with a lesser effect upon withdrawal of the drug. Similar situations are reported in rheumatoid arthritis patients treated with the *TNF*-α antagonist etanercept, or even in solid organ transplant patients under a high dose of immunosuppression [9,10]. Immunomodulatory agents interfere with the balance between the varying components of the immune system. When combined with the drug induced release of viral particles in hepatitis C or self antigens in autoimmune diseases, they seem to be sufficient to induce sarcoidosis.

Sarcoidosis seems to be a shared outcome of different triggering factors in a susceptible host, rather than a homogenous disease with a specific aetiology. We propose here two synergistic mechanisms in the formation of exuberant non-caseating granulomas that define sarcoidosis. This includes T cell dysfunction and exposure to antigens, not readily degradable by other means, regardless of whether they are foreign, tumour associated or self antigens. This hypothesis, which we basically predict from our observations and from the limited knowledge in the literature, needs to be verified by further research.

## Conclusions

In our patients, immunosuppression intrinsic to their lymphoma, accentuated by chemotherapy, superimposed with the release of abundant tumour antigens may explain the formation of sarcoidosis. In the patient with HL, we hypothesize that sarcoidosis might be the underlying reason for the unexplained respiratory difficulty experienced during treatment. Release of abundant tumour antigens during chemotherapy might have triggered a sarcoidal reaction which was initially suppressed by the ongoing therapy and became clinically evident thereafter.

## Abbreviations

ABVD: doxorubicin, bleomycin, vinblastine, and dacarbazine; EBV: Epstein-Barr virus; FDG: fluorodeoxyglucose; HL: Hodgkin's lymphoma; LN: lymph node; PET-CT: positron emission tomography-computed tomography; R-CHOP: rituximab, cyclophosphamide, doxorubicin, vincristine, prednisolone; RS: Reed-Sternberg.

## Consent

Written informed consent was obtained from the patients for publication of these case reports and any accompanying images. A copy of the written consent is available for review by the Editor-in-Chief of this journal.

## Competing interests

The authors declare that they have no competing interests.

## Authors' contributions

OO performed the histopathological examination of the lymph nodes and bone marrow, and was a major contributor in writing the manuscript. MCA, HB, AEE, and BF interpreted the patients' data in terms of haematological disease; MCA and BF also took part in writing the manuscript. FT analysed and interpreted the patients' data regarding the infectious lung disease. GO analysed and interpreted the patients' data and clinical findings related to sarcoidosis. All authors read and approved the final manuscript.
